# Location, length, and enhancement: systematic approach to differentiating intramedullary spinal cord lesions

**DOI:** 10.1007/s13244-018-0608-3

**Published:** 2018-06-12

**Authors:** Sarah Mohajeri Moghaddam, Alok A. Bhatt

**Affiliations:** Department of Imaging Sciences, University of Rochester Medical Center, 601 Elmwood Avenue, P.O. Box 648, Rochester, NY 14642 USA

**Keywords:** Spinal cord, Demyelinating diseases, Spinal neoplasms, MR, Review

## Abstract

**Purpose:**

Intramedullary spinal cord abnormalities are often challenging to diagnose. Spinal cord biopsy is a high-risk procedure with the potential to cause permanent neurological injury. Magnetic resonance imaging is the modality of choice for diagnosis and preoperative assessment of patients with spinal cord abnormalities. The radiologist’s ability to narrow the differential diagnosis of spinal cord abnormalities has the potential to save patients from invasive approaches for diagnosis and also guide appropriate management.

**Approach/methods:**

This article will provide a systematic approach to the evaluation of intramedullary spinal cord lesions—with emphasis on location, length and segment distribution, and enhancement pattern—to help narrow the differential diagnosis. In doing so, we will review various spinal cord pathologies, including demyelinating and metabolic conditions, neoplasms, and vascular lesions.

**Summary/conclusion:**

Although intramedullary spinal cord abnormalities can be a challenge for the radiologist, a systematic approach to the differential diagnosis with a focus on lesion location, cord length and segment involvement, as well as enhancement pattern, can greatly help narrow the differential diagnosis, if not synch the diagnosis. This strategy will potentially obviate the need for an invasive approach to diagnosis and help guide treatment.

**Teaching points:**

• *Imaging diagnosis of intramedullary spinal cord lesions could obviate cord biopsy.*

• *Evaluation of cord lesions should focus on location, length, and enhancement pattern.*

• *In demyelination, the degree of cross-sectional involvement is a distinguishing feature.*

## Introduction

Intramedullary spinal cord abnormalities are a diagnostic challenge. Spinal cord biopsy is a high-risk procedure with potential to cause permanent neurological injury [1]. Magnetic resonance imaging (MRI) is the modality of choice for diagnosis and preoperative assessment of patients with spinal cord abnormalities. The radiologist’s ability to narrow the differential diagnosis of spinal cord abnormalities has the potential to save patients from invasive approaches to diagnosis and guide appropriate management. This article will provide a systematic approach for the evaluation of intramedullary spinal cord lesions to help narrow the differential diagnosis, if not synch the diagnosis.

**Fig. 1:**
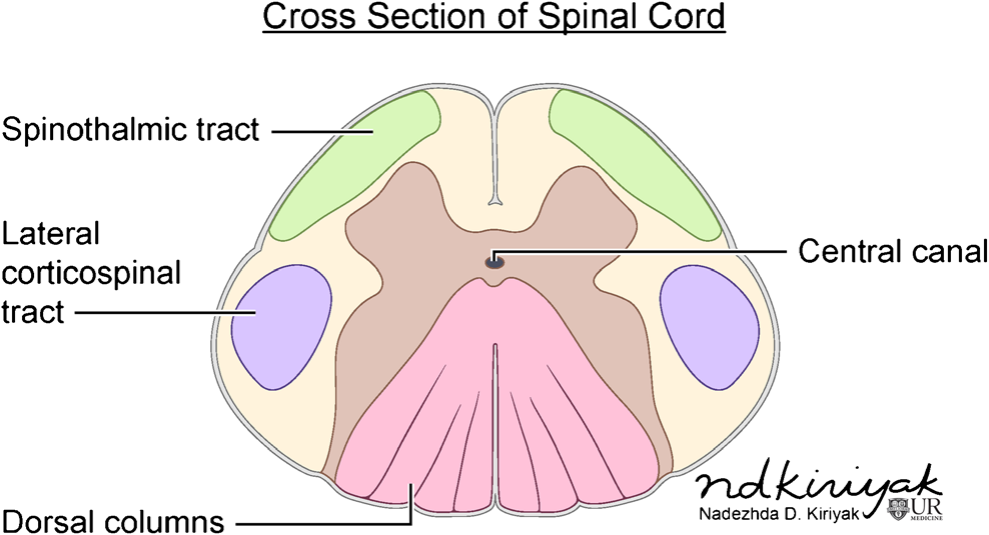
Intramedullary spinal cord anatomy

Knowledge of spinal cord anatomy is essential for thorough evaluation. The spinal cord extends caudally from the brainstem to the conus medullaris at about the L1–L2 vertebral level. It consists of 31 levels, which are divided into the cervical (8 nerve roots), thoracic (12 nerve roots), lumbar (5 nerve roots), sacral (5 nerve roots), and coccygeal (1 nerve root) levels where nerve roots emerge. As within the brain, the spinal cord is covered by three layers of the meninges: the outer dura mater, middle arachnoid mater, and inner pia mater. Intramedullary anatomy consists of white matter in the form of myelinated ascending and descending fibres and includes the anterior ascending pain and temperature sensory fibres of the spinothalamic tracts, dorsal columns containing ascending vibration and proprioception fibres, and lateral columns, which contain the descending corticospinal tract fibres (Fig. 1). The central grey matter consists of anterior horns containing motor neurons that synapse with the descending corticospinal tract fibres and posterior horns, which are composed of sensory neurons that synapse with ascending sensory fibres [2, 3]. Spinal pathology can be divided into three general categories based on spatial localization: the extradural space, intradural-extramedullary space, and intramedullary space. This article will focus on pathology within the intramedullary space.

**Fig. 2:**
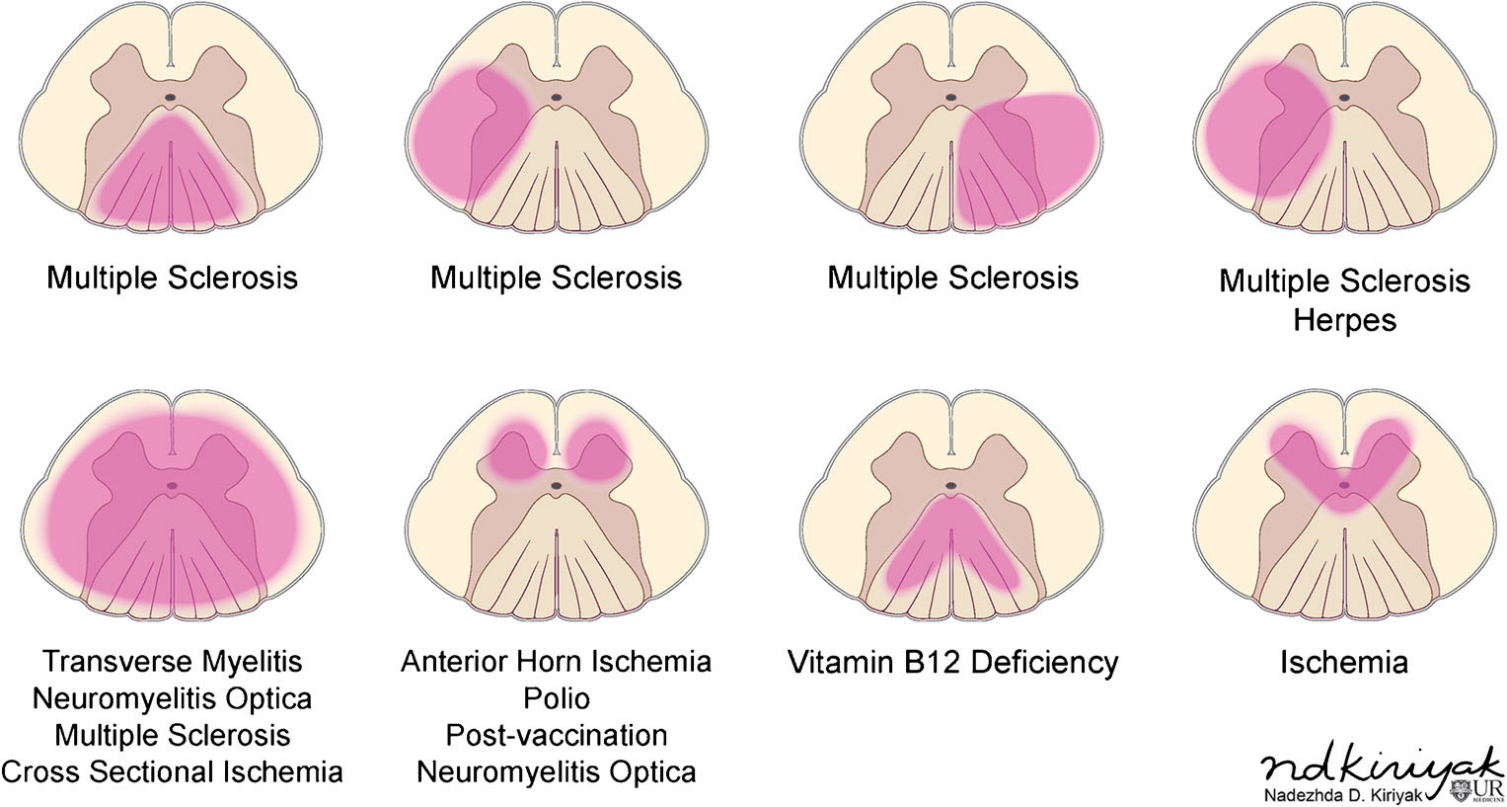
Differentiating intramedullary pathology: location within the cord

**Fig. 3:**
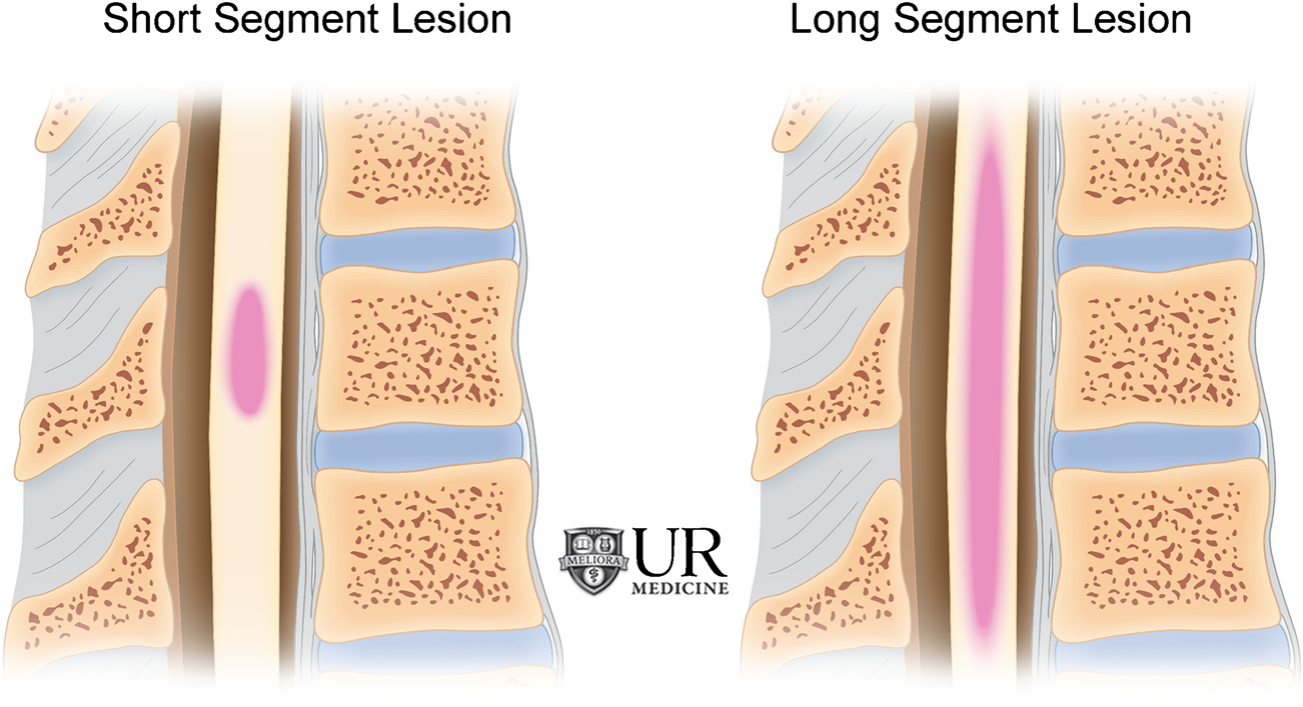
Differentiating intramedullary pathology: short segment (left) versus long segment (right) lesions

Differentiating intramedullary pathology can be challenging given the spinal cord’s morphology and surrounding osseous and ligamentous structures. However, with the advent of MRI, the differential diagnosis can be narrowed by careful analysis of the pattern of involvement, with particular attention given to the location, length, and enhancement pattern. Transverse location, for example, is important to consider when differentiating demyelinating processes (Fig. 2), while location along the cord can help narrow tumor diagnoses. Segmental length of involvement (Fig. 3) can be helpful in differentiating demyelinating processes or help distinguish between neoplastic and vascular lesions. Lastly, presence of multiplicity and the pattern of enhancement should be considered.

## Demyelination

### Multiple sclerosis

**Fig. 4:**
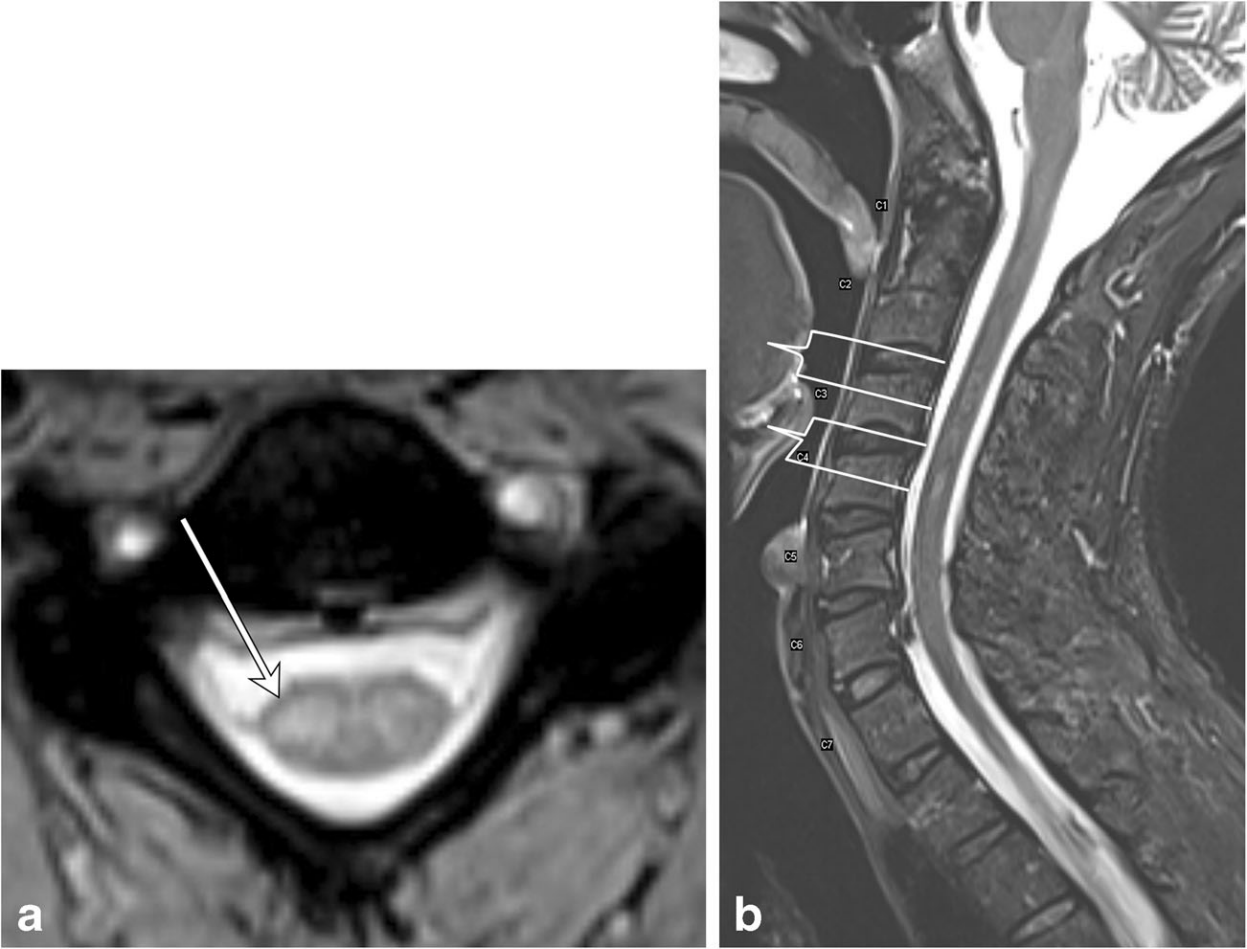
Multiple sclerosis. A 50-year-old male with a history of multiple sclerosis. **a** Axial MERGE image demonstrates partial cord involvement—focal hyperintensity within the right lateral cord with a characteristic triangular shape (arrow). There is no cord enlargement. **b** Sagittal T2 fat-saturated image demonstrates several scattered, short-segment foci of T2 hyperintensity (brackets)

**Fig. 5:**
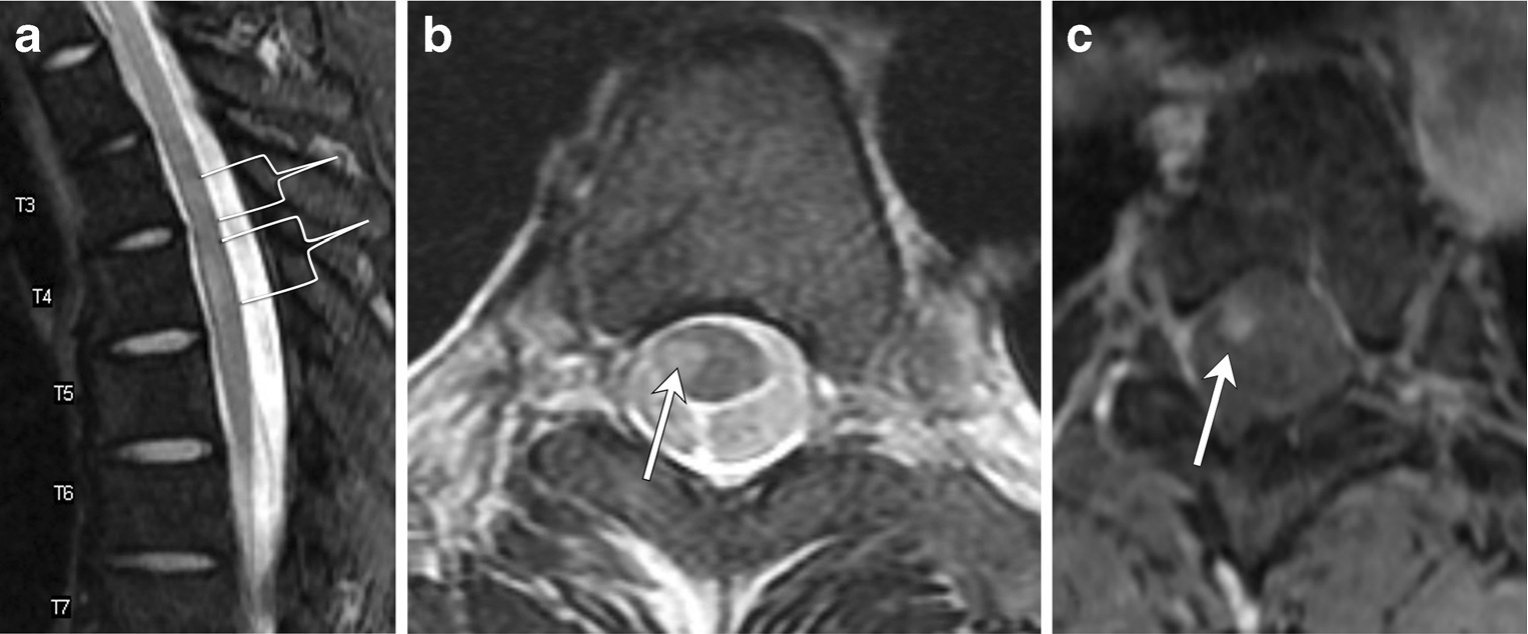
Multiple sclerosis. A 50-year-old female with prior history of visual changes, presenting with right extremity weakness. **a** Sagittal STIR image shows several foci of short segment hyperintensity (brackets). **b** Axial T2 image demonstrates partial cord involvement, with a triangular lesion involving the right lateral cord (arrow). **c** Axial T1 post-contrast image demonstrates associated enhancement (arrow), consistent with active demyelination

In demyelinating conditions, the underlying mechanism of myelopathy is that of inflammation damaging myelin sheath-forming cells. This can be primary, due to conditions such as multiple sclerosis (MS), or secondary, such as in post-infectious acute disseminated encephalomyelitis (ADEM) or transverse myelitis secondary to underlying viral infection [4]. MS is a primary demyelinating disease affecting the central nervous system with intracranial and spinal involvement (Figs. 4 and 5). The disease is twice as common in females and tends to occur in geographical regions farther away from the equator. MRI is the most important modality in both the diagnosis and clinical management of MS, demonstrating characteristic callososeptal and periventricular, perivenular demyelinating lesions in the brain [5]. Spinal cord involvement of MS frequently occurs along with brain involvement, although isolated spinal cord lesions can occur in 25% of patients. In Asian populations, who are more likely to present with the optic-spinal variant of MS, spinal cord involvement occurs more frequently, and the cord should be carefully evaluated in this subset of the population [6].

### Neuromyelitis optica spectrum disease (NMOSD)

**Fig. 6:**
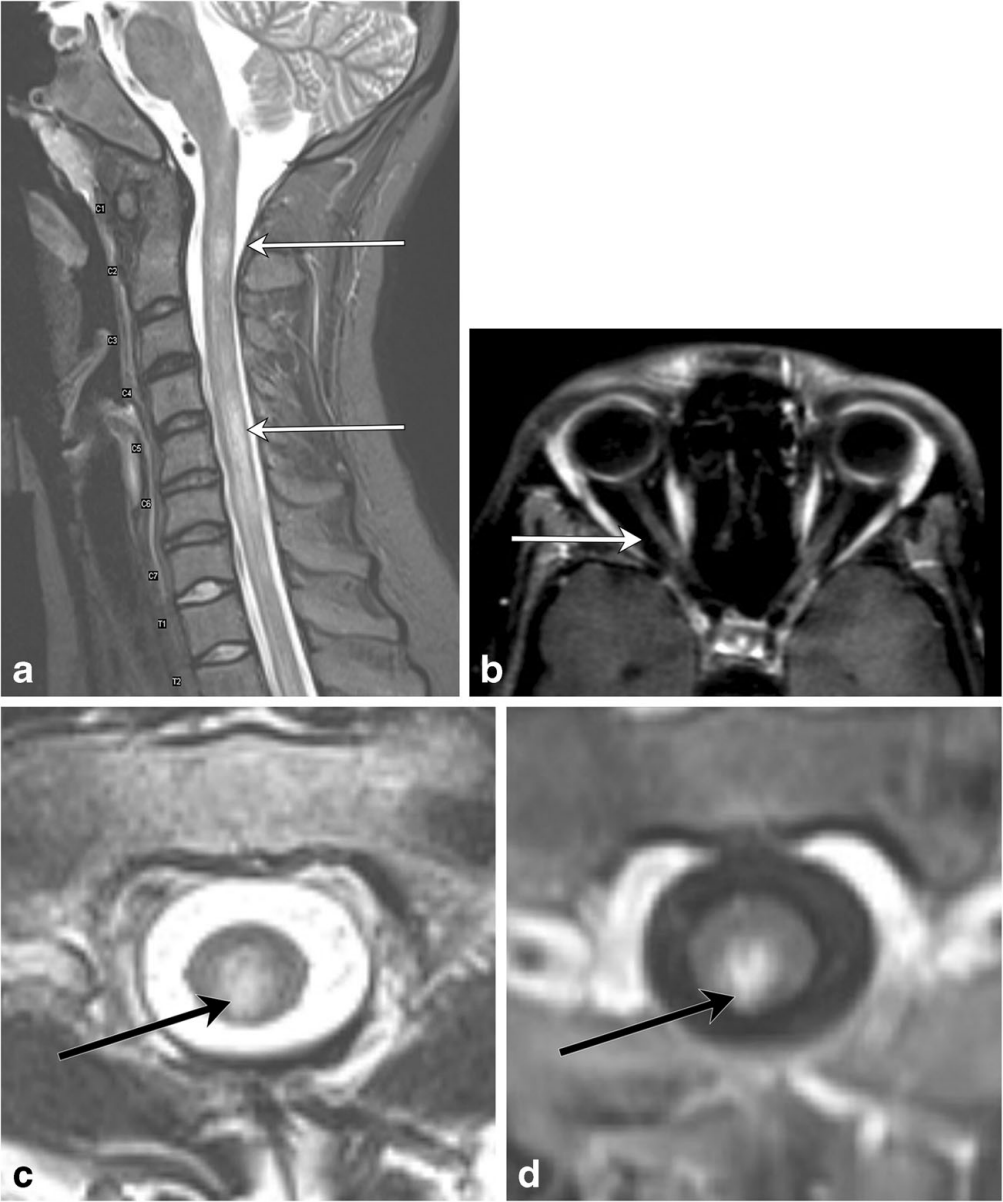
Neuromyelitis optica spectrum disease. A 20-year-old female presents with lower back pain and difficulty walking. **a** Sagittal T2 fat-saturated image demonstrates multiple areas of long-segment hyperintensity (arrows). **b** Axial T1 post-contrast image of the orbits reveals subtle right optic nerve enhancement (arrow). **c** Axial T2 image shows central cord involvement (arrow). **d** Axial T1 post-contrast image demonstrates corresponding enhancement (arrow)

NMOSD (also known as Devic’s disease) is a relapsing disease with the typical triad of optic neuritis, myelitis, and positive NMO-IgG, an autoantibody to the protein channel aquaporin-4 (AQP4), which is expressed on foot processes of astrocytes (Fig. 6). The underlying pathophysiology is of autoimmune astrocytopathy [7], distinguishing this condition from MS and other demyelinating processes. The condition is much more common in females (5:1), and brain lesions are shown to occur much more frequently than previously thought. Among other diagnostic criteria, presence of longitudinally extensive spinal cord lesions (LESCL), which are characterised by T2 hyperintense signal of the cord traversing at least three vertebral body levels, is key to its diagnosis [8, 9].

### Acute disseminated encephalomyelitis

**Fig. 7:**
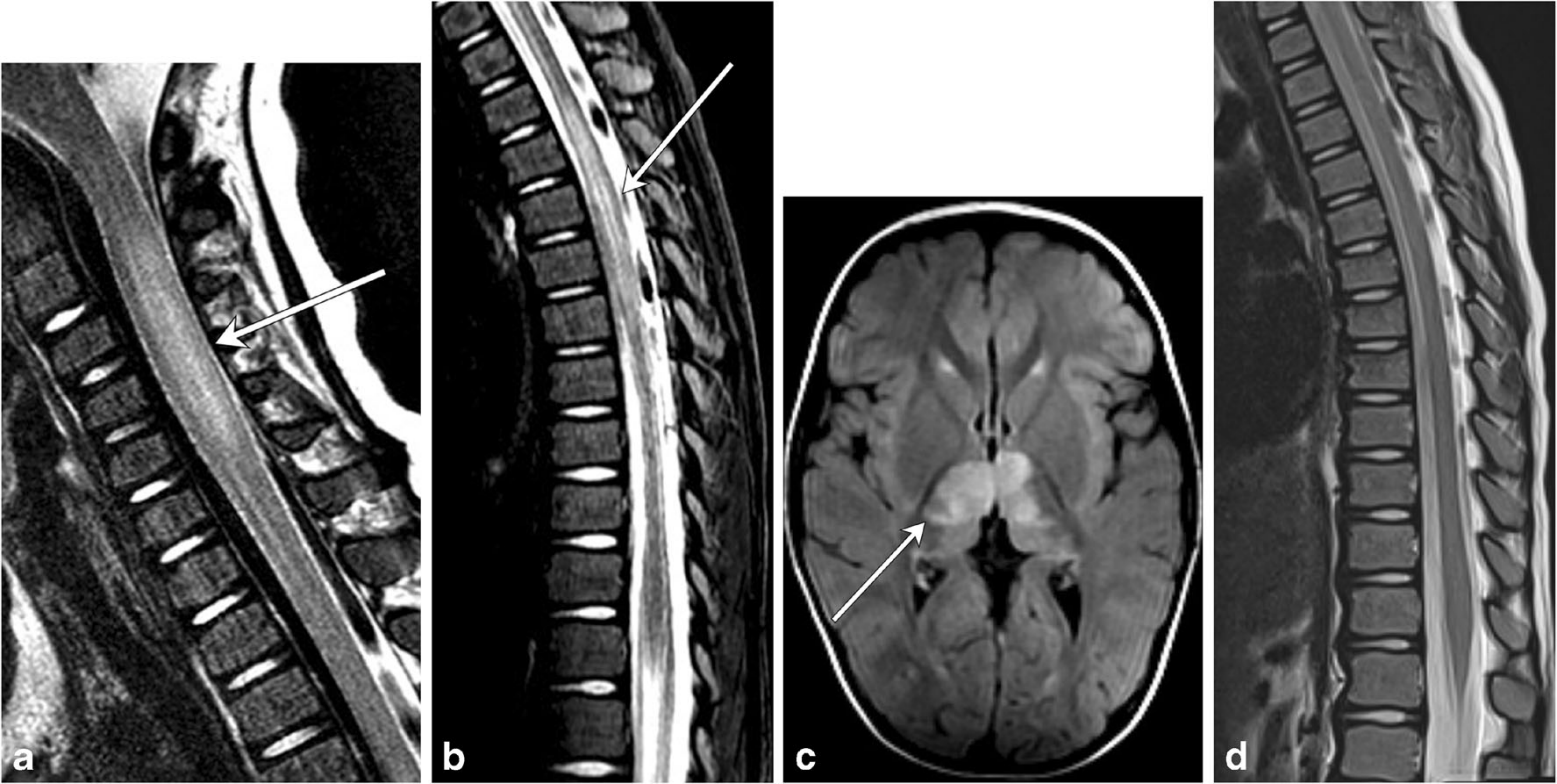
Acute disseminated encephalomyelitis (ADEM). A 2-year-old boy with recent upper respiratory tract infection and low-grade fevers presents with lower extremity weakness and difficulty walking. **a** and **b** Sagittal T2 images of the cervical and thoracic spine demonstrate long-segment areas of hyperintensity involving the cervical and thoracic spinal cord (arrows). There is mild associated cord swelling. **c** Axial T2 FLAIR image shows mass-like hyperintensity within the thalami (arrow). **d** Follow-up sagittal T2 image of the thoracic spine demonstrates resolution of the previously seen lesions—normal cord

ADEM is an acute, often monophasic post-infectious or inflammatory condition that most commonly occurs in children (Fig. 7). ADEM can involve both white and grey matter, and although it can demonstrate periventricular brain lesions similar to MS, it is more likely to have ill-defined, larger, and more rounded lesions, with a predilection for deep grey matter and the brainstem [10, 11]. Involvement of the brainstem and spinal cord occurs in approximately one-third of patients with ADEM and its spinal characteristics are helpful in differentiating this condition from MS [5].

### Guillain-Barré syndrome

**Fig. 8:**
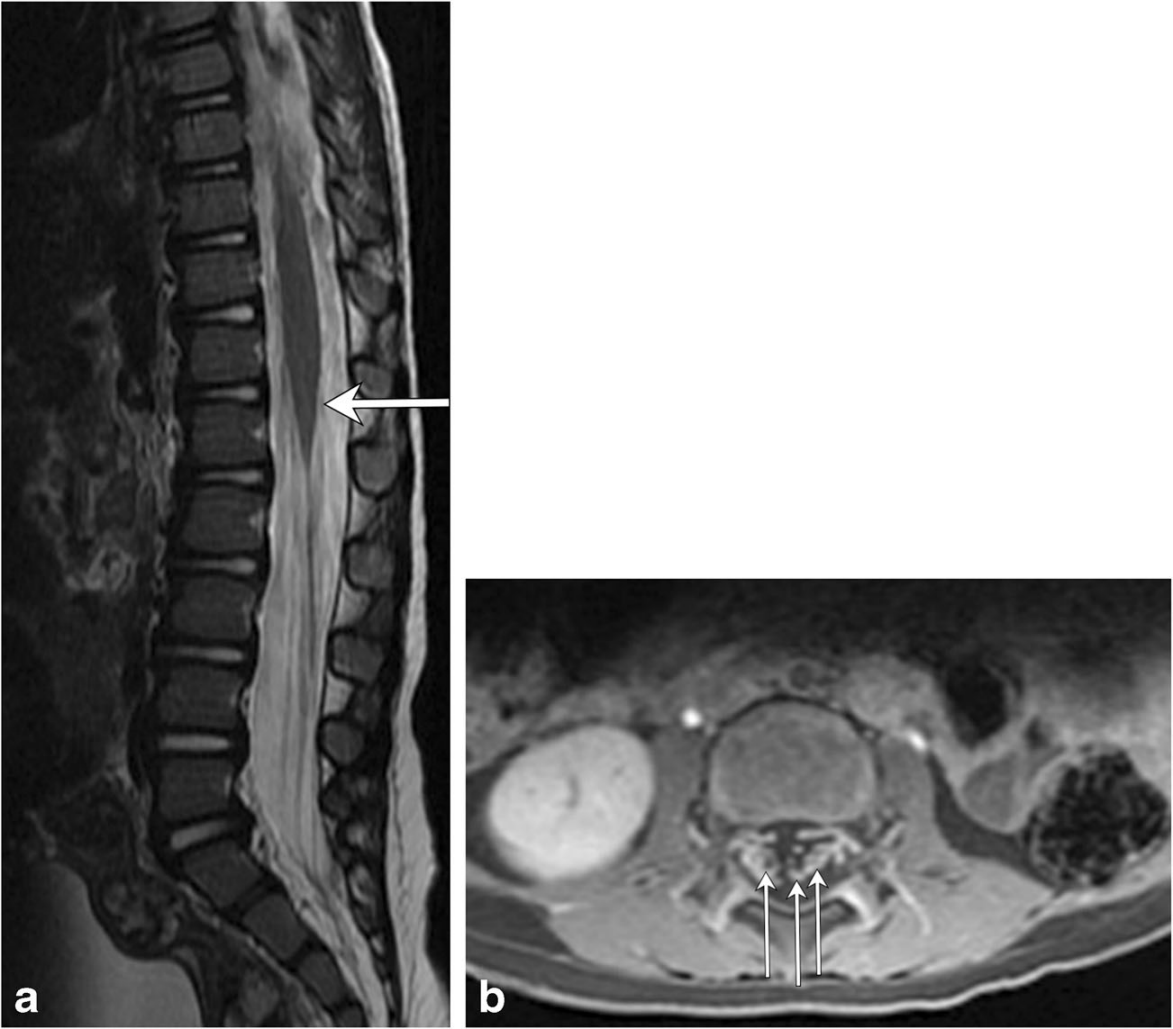
Guillian-Barré syndrome. A 2-year-old patient status post-viral prodrome with progressive ascending weakness/paralysis. **a** Sagittal T2 image of the thoracolumbar spine demonstrates subtle hyperintensity at the level of the conus medullaris (arrow). **b** Axial T1 post-contrast image of the lumbar spine demonstrates enhancement of multiple nerve roots (arrows)

Guillain-Barré syndrome (GBS) can be thought of as the peripheral nervous system counterpart to ADEM, with a post-infectious/inflammatory autoimmune pathophysiology (Fig. 8). It typically involves multiple peripheral nerves, most commonly the anterior nerve roots arising at the cauda equina. The classic clinical history consists of ascending weakness, which can progress to ascending paralysis, typically occurring after a bout of infection or after having received a vaccination. Most cases resolve within a few weeks without residual symptoms [12, 13].

### Transverse myelitis

**Fig. 9:**
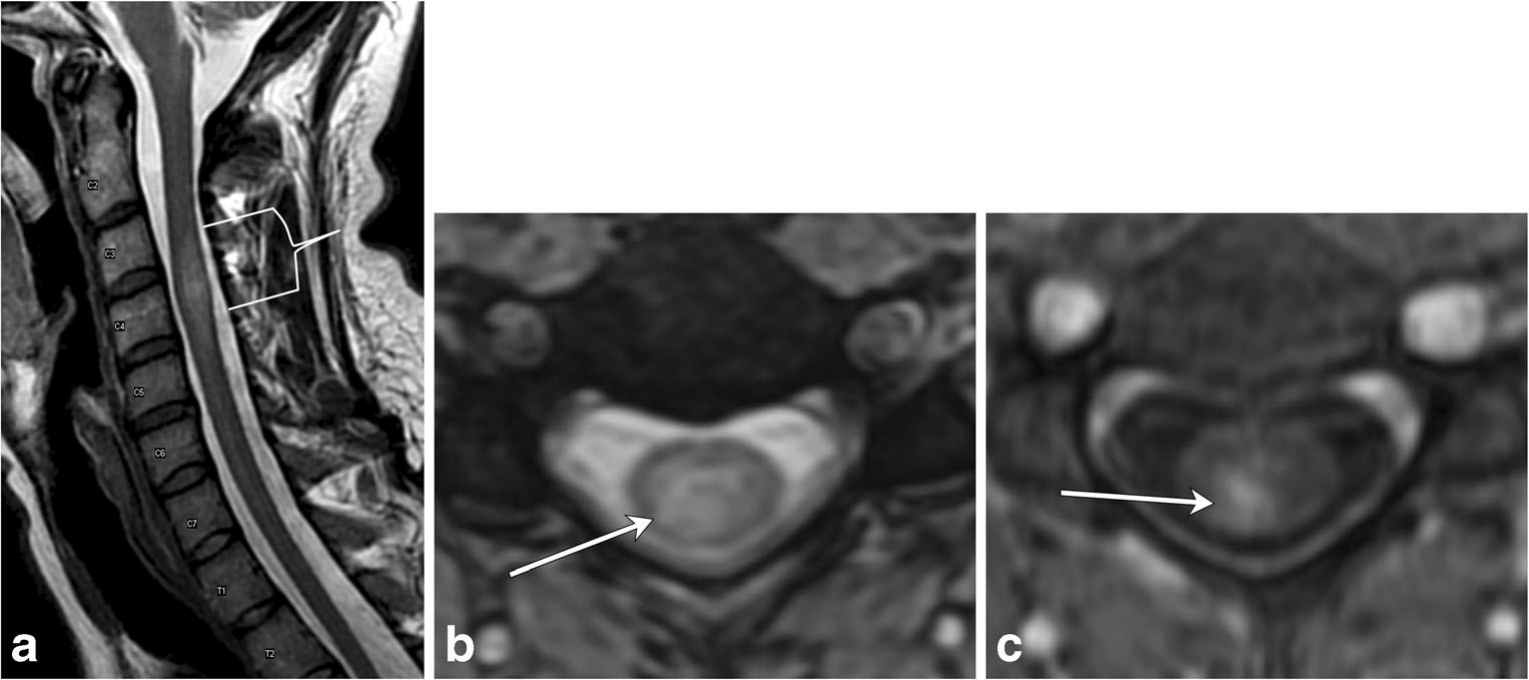
Transverse myelitis. A 38-year-old female with progressive bilateral extremity and back numbness. **a** Sagittal T2 image demonstrates short segment hyperintensity (bracket) with slight expansion of the cervical cord **b** Axial T2 image demonstrates greater than 2/3 cord involvement (arrow). c T1 post-contrast image demonstrates corresponding dusky enhancement

Transverse myelitis is an inflammatory condition of the spinal cord with various underlying aetiologies. This condition may occur at any age; however, it is more common in younger patients. It typically presents with rapidly progressive bilateral sensory and motor dysfunction with a distinct cord level. Approximately one-third of affected patients recover fully while the remaining 60 % will have either moderate or profound neurological deficits. There are various underlying aetiologies of transverse myelitis, including MS, systemic autoimmune disorders such as systemic lupus erythematosus, vascular infarct, post-radiation, post-infectious, or idiopathic. MRI demonstrates a long segment T2 hyperintense cord lesion with bilateral cord involvement, occupying at least two-thirds of the cross-sectional area of the cord (Fig. 9) [14, 15].

### HIV myelopathy

**Fig. 10:**
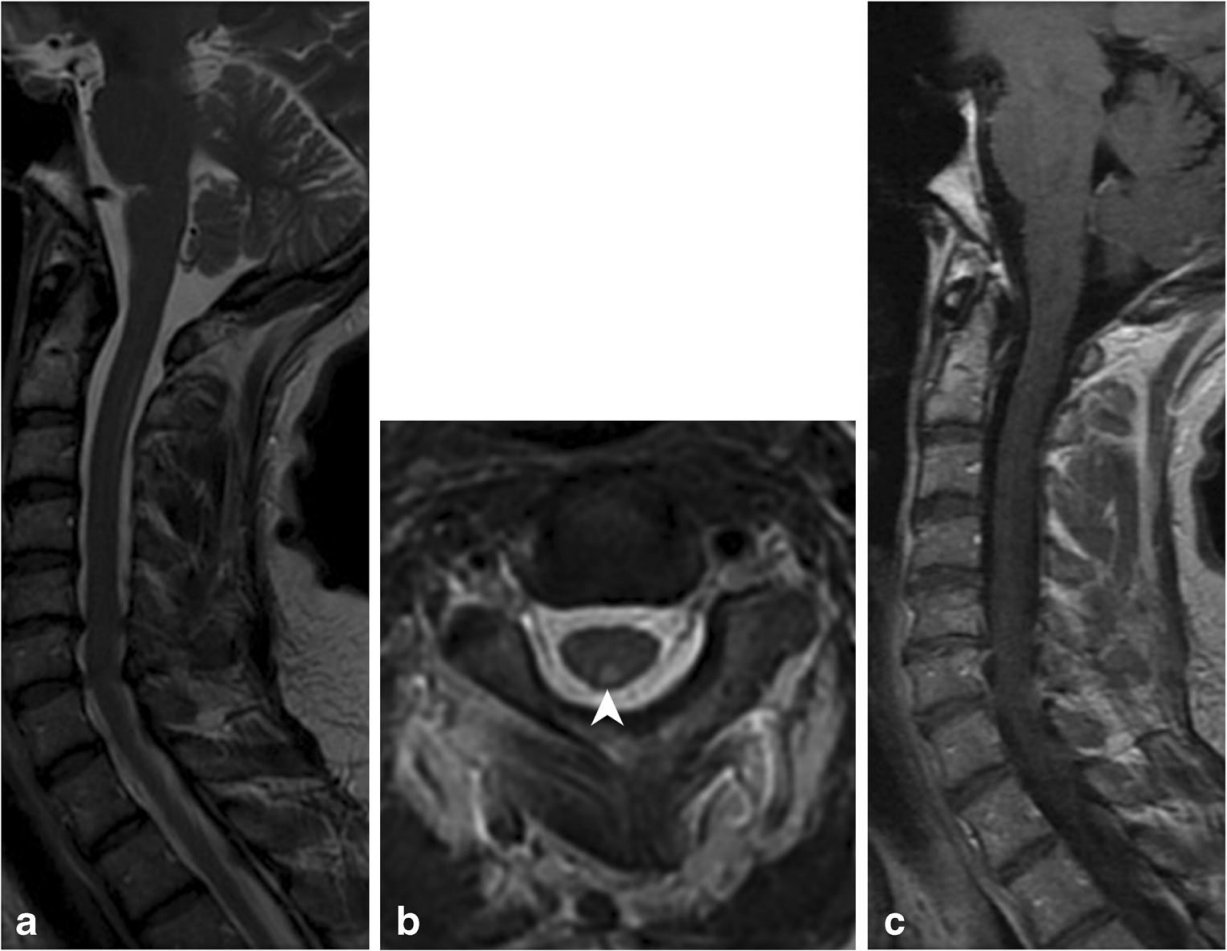
HIV myelitis. A 54-year-old female with a history of HIV presents with peripheral neuropathy. **a** Sagittal T2-weighted image demonstrates a long segment of hyperintensity in the dorsal cord. **b** Axial T2-weighted image shows a well-defined dorsal cord lesion (arrowhead) with normal signal within the remainder of the cord. **c** Sagittal T1 post- contrast image shows no enhancement

HIV myelopathy is a late-onset complication of HIV (Fig. 10). Patients present with insidious onset myelopathy with lower extremity weakness and sensory abnormalities, impotence, and/or urinary symptoms. On MRI, spinal cord atrophy is typically present. Other findings can be seen, including symmetric dorsal column hyperintensity on T2-weighted images similar to subacute combined degeneration, though typically the signal abnormality is limited to the thoracic spine [16, 17]. The cervical spine is involved less commonly. On histology, there is vacuolization of the myelin-forming cells of the spinal cord [17].

### Distinguishing demyelinating conditions

In differentiating spinal demyelinating processes, the location along the spine and degree of transverse cord involvement can be distinguishing features. In MS, lesions are more commonly located within the cervical spine and are often multifocal and asymmetric, involving a partial cross-sectional area of the cord, for example, involving a lateral or posterior aspect of the cord on an axial slice. This is in contrast to NMOSD and transverse myelitis, where there is central or more diffuse cord involvement on axial images [6]. MS lesions involve short segments (1–2 vertebral bodies), whereas in NMOSD, long segment and central involvement is more characteristic [11]. MS lesions appear more homogeneous, whereas NMOSD lesions tend to be more ill defined and heterogeneous in T2 hyperintensity [5]. ADEM will demonstrate diffuse T2 hyperintensity and expand the spinal cord; a long segment is commonly involved, and there is no enhancement [18]. If myelopathy is isolated to the conus medullaris/cauda equina, one must consider GBS, in which there is nerve root enhancement, particularly involving the anterior nerve roots [11]. Enhancing lesions in the cord may be seen with active demyelination in MS and NMOSD. The pattern of enhancement is variable with transverse myelitis, while ADEM lesions most commonly do not enhance. Ring-enhancing lesions, which are characteristically seen with tumefactive MS in the brain, can also rarely be present in the spinal cord. In NMOSD, a distinctive ‘lens-shaped’ pattern of enhancement on sagittal images is characteristic if present [18] (Table 1).

**Table 1:** Demyelinating disease summary

Demyelinating diseases	Location	Length and segment distribution	Enhancement
Multiple sclerosis	Dorsal or lateral columns	Short segment, scattered	Yes, if active
NMOSD (Devic’s disease)	Central	Long segment	Patchy
ADEM	Diffuse swelling of cord	Long segment	None
Guillian-Barré	Diffuse Nerve roots	Conus medullaris, cauda equina roots	Yes
Transverse myelitis	> 2/3 on axial view; swelling of cord	Short (acute partial) or long (acute complete)	Variable

### Metabolic conditions

**Fig. 11:**
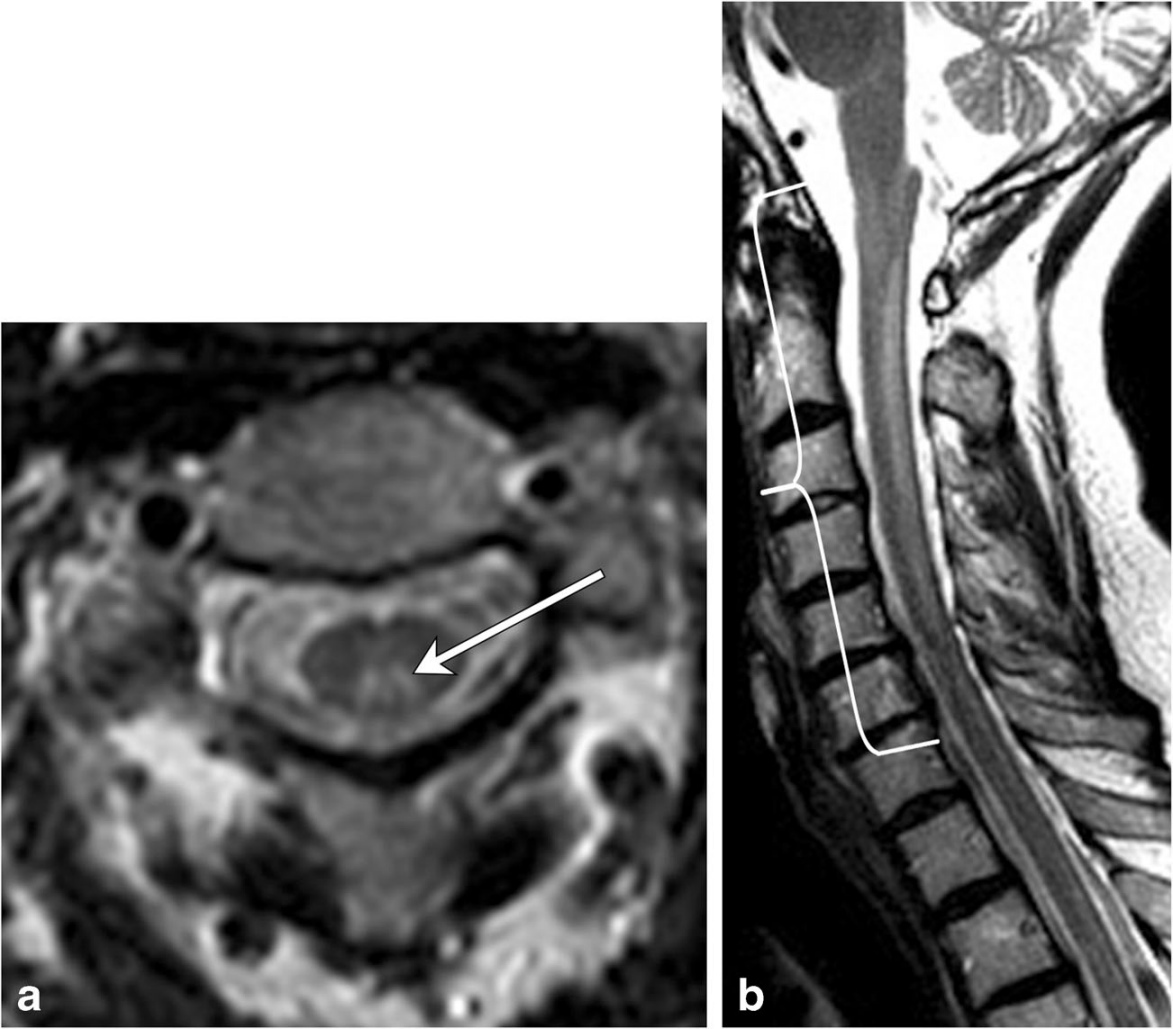
Subacute combined degeneration due to copper deficiency. A 45-year-old female with tingling in the bilateral upper and lower extremities. **a** Axial T2 image shows hyperintensity in the dorsal columns in an ‘inverted-V’ configuration (arrow). **b** Sagittal T2 image demonstrates long-segment T2 hyperintensity within the dorsal cord (bracket)

**Fig. 12:**
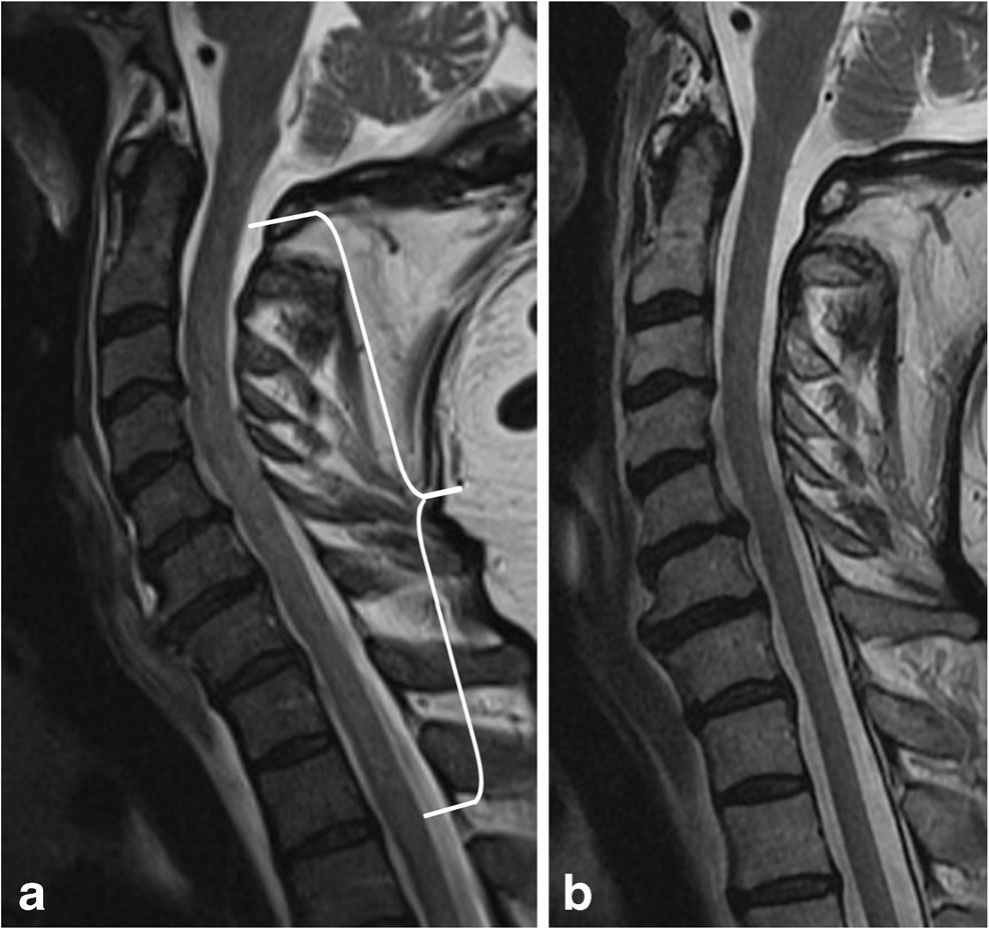
Subacute combined degeneration due to vitamin B12 deficiency secondary to pernicious anemia. A 58-year-old female with tingling in her hands and feet. **a** Pre-treatment sagittal T2 image demonstrates a long segment of high signal in the dorsal cord (bracket). **b** Post-treatment sagittal T2 image demonstrates resolution of the cord signal abnormality

Subacute combined degeneration is the most common manifestation of metabolic derangements within the spinal cord. The condition presents clinically with loss of vibration and proprioception in the extremities, eventually leading to gait ataxia and extremity muscle weakness. The aetiology may be due to copper deficiency (Fig. 11) or nitric oxide use; however, the most common aetiology is vitamin B12 deficiency (Fig. 12). On MRI, the characteristic pattern is of long segment T2 hyperintensity involving the posterior spinal cord, classically involving the dorsal columns and producing a characteristic reversed “V” sign [19, 20] (Table 2).

**Table 2:** Metabolic condition summary

Metabolic	Location	Length and segment distribution	Enhancement
Subacute combined degeneration • Vitamin B12 deficiency • Copper deficiency • Nitric oxide inhalation	Dorsal columns	Long segment	None

## Neoplasms

### Ependymoma

**Fig. 13:**
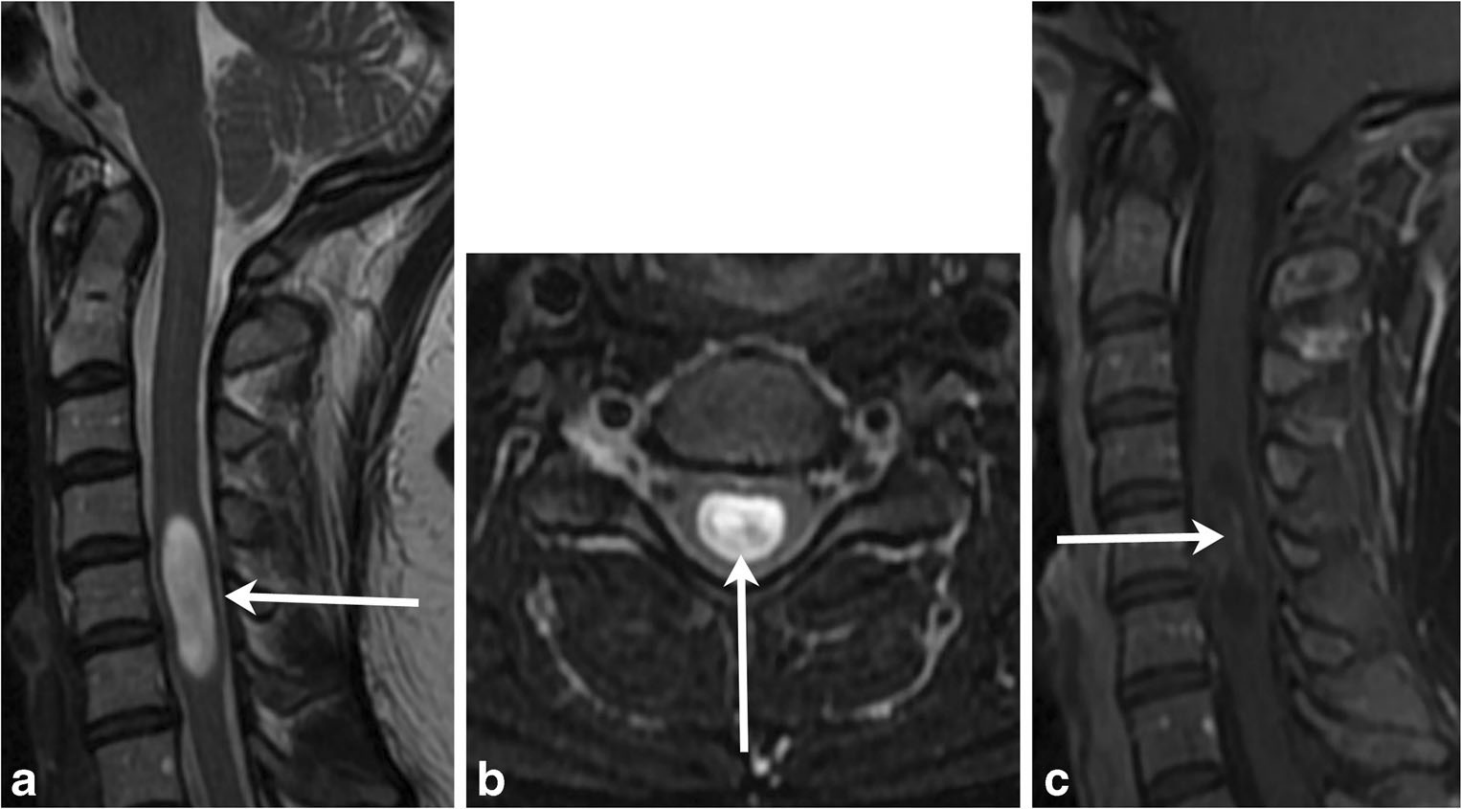
Ependymoma. A 38-year-old female with pulse-like sensation in both hands. **a** and **b** T2 hyperintense mass (arrow) involves more than two vertebral body levels within the central cord. **c** Minimal heterogeneous enhancement (arrow) on the T1 post-contrast image

Ependymomas are benign glial tumors that arise from ependymal cells lining the brain’s ventricles or the central canal of the spinal cord (Fig. 13). Although the majority of ependymomas occur intracranially, about 10% occur in the spinal cord and represent the most common intramedullary spinal cord tumor in adults. Spinal ependymomas most commonly occur in the cervical cord, though they can occur anywhere along the spinal cord. The myxopapillary subtype is confined to the filum terminale with rare extension into the conus medullaris. Their association with neurofibromatosis type II is explained by abnormality found within chromosome 22; the NF2 gene is located on chromosome 22q and patients with an ependymoma often have defects on the same chromosome [21–24].

### Hemangioblastomas

**Fig. 14:**
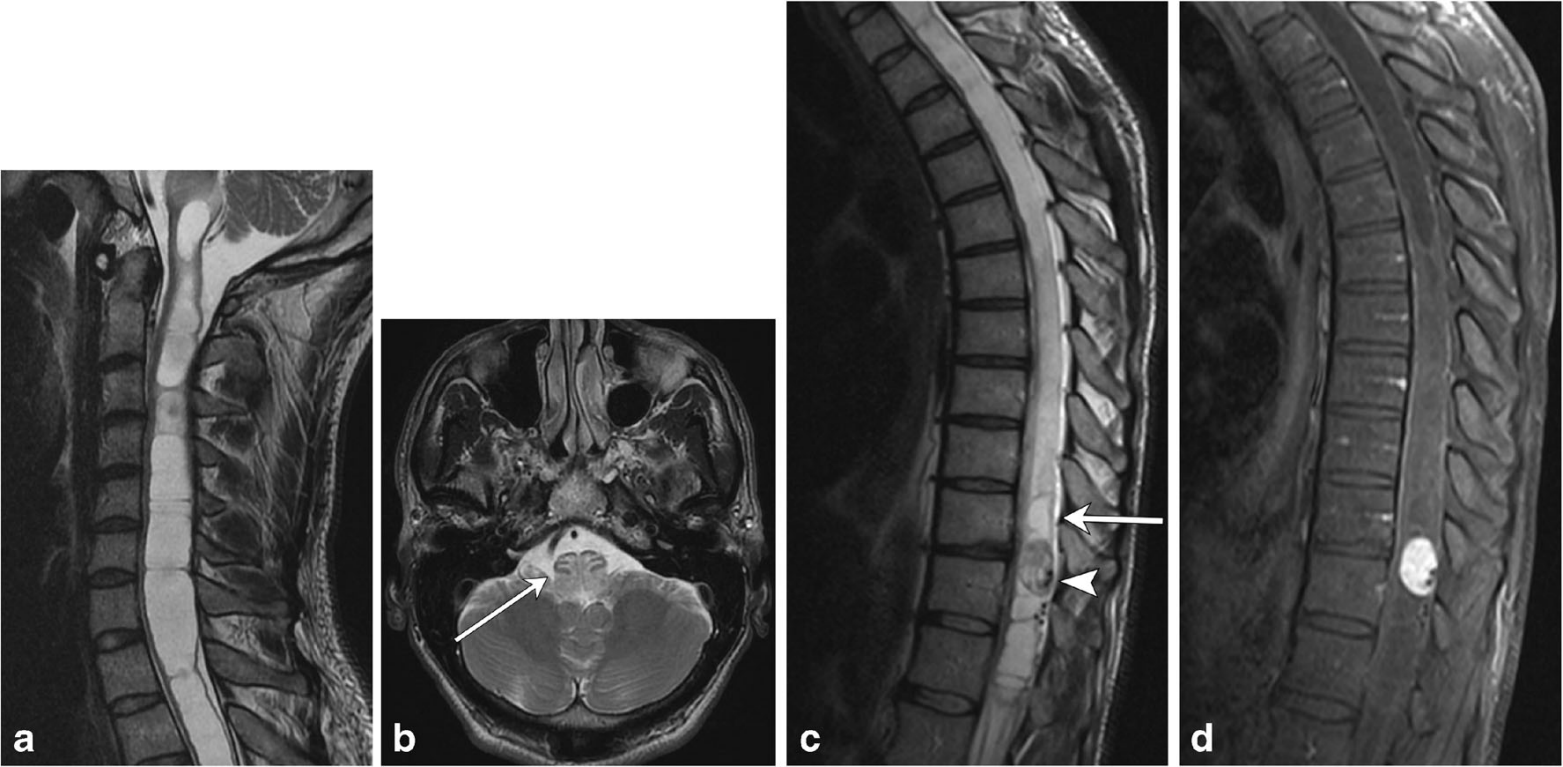
Hemangioblastoma. A 40-year-old male with neck spasms and gait disturbance. **a** and **b** Spinal cord syrinx involving the entirety of the cervical cord extends superiorly to the cervicomedullary junction, where there is mass effect on the brainstem with resulting edema (arrow); therefore, more inferior spine imaging was obtained to look for the cause. **c** Sagittal T2 image shows an intramedullary lesion within the lower thoracic cord with associated syrinx (arrow). Serpiginous flow voids (arrowhead) are feeding vessels. **d** T1 post-contrast fat-saturated image demonstrates a nodule of homogeneous enhancement

Hemangioblastomas are rare benign tumors that most commonly occur in the cerebellum and represent the third most common intramedullary spinal cord tumor (Fig. 14). They are associated with Von Hippel Lindau syndrome (VHL), particularly when seen within the cord, or if multiple lesions are detected. They are thought to arise from undifferentiated mesenchymal cells and are composed of densely packed capillaries lined with endothelial cells. There is no gender predilection, and they most commonly arise in the thoracic cord followed by the cervical cord [21–23].

### Astrocytoma

**Fig. 15:**
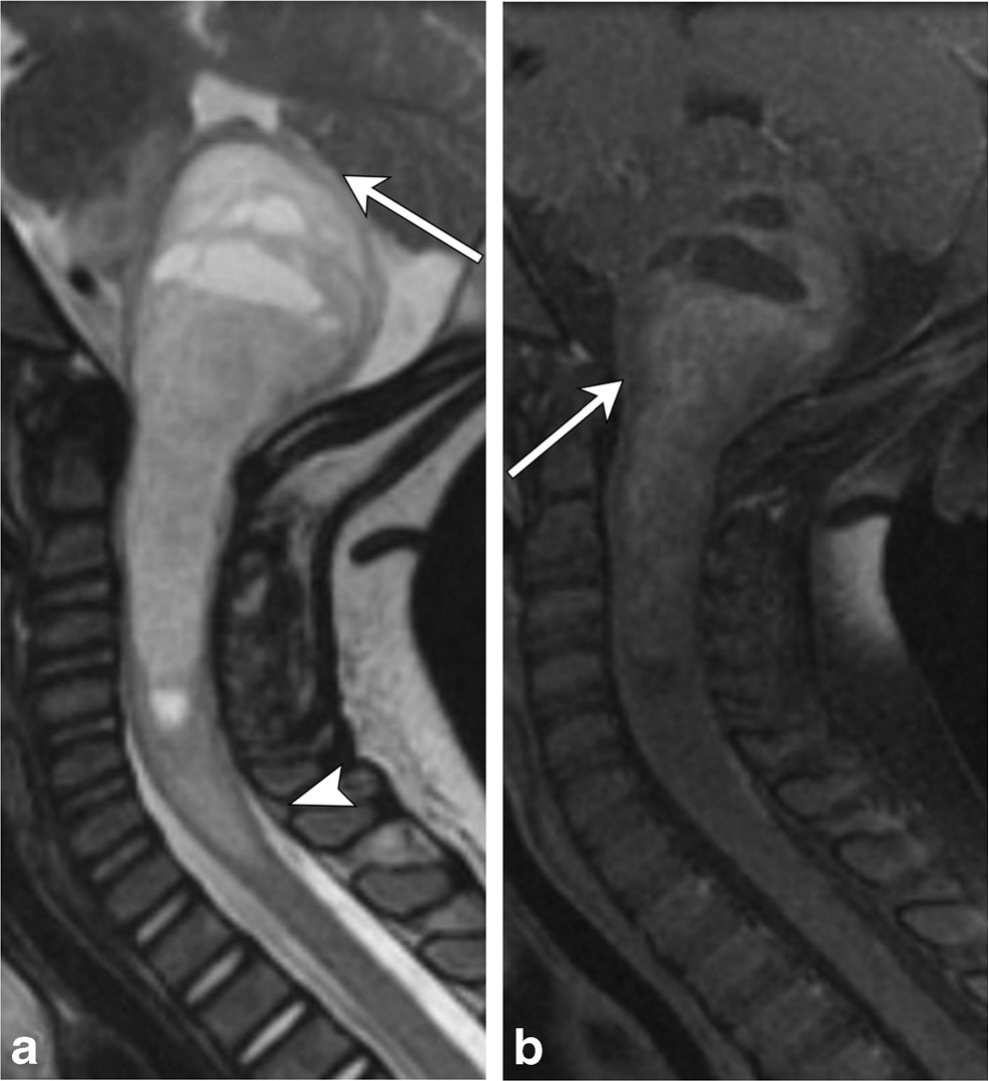
Spinal astrocytoma. A 3 year old with a history of torticollis and ear pain. **a** Sagittal T2 image demonstrates a heterogeneous, expansile solid and cystic mass involving the upper cervical spine and brainstem, compressing the fourth ventricle and cerebellum (arrow). Mass/edema extends inferiorly to the C7-T1 vertebral level (arrowhead). **b** There is heterogeneous enhancement of the solid components on the T1 post-contrast image (arrow)

Intramedullary astrocytomas are most common in children and tend to occur more frequently in males (Fig. 15). They are likely to occur in the thoracic cord, followed by the cervical cord, involve multiple vertebral body levels and are infiltrative in nature. They are associated with neurofibromatosis type I. High-grade intramedullary astrocytomas are very rare, though glioblastoma multiforme has been reported, with which leptomeningeal involvement is commonly present [22–24].

### Distinguishing intramedullary tumors

**Fig. 16:**
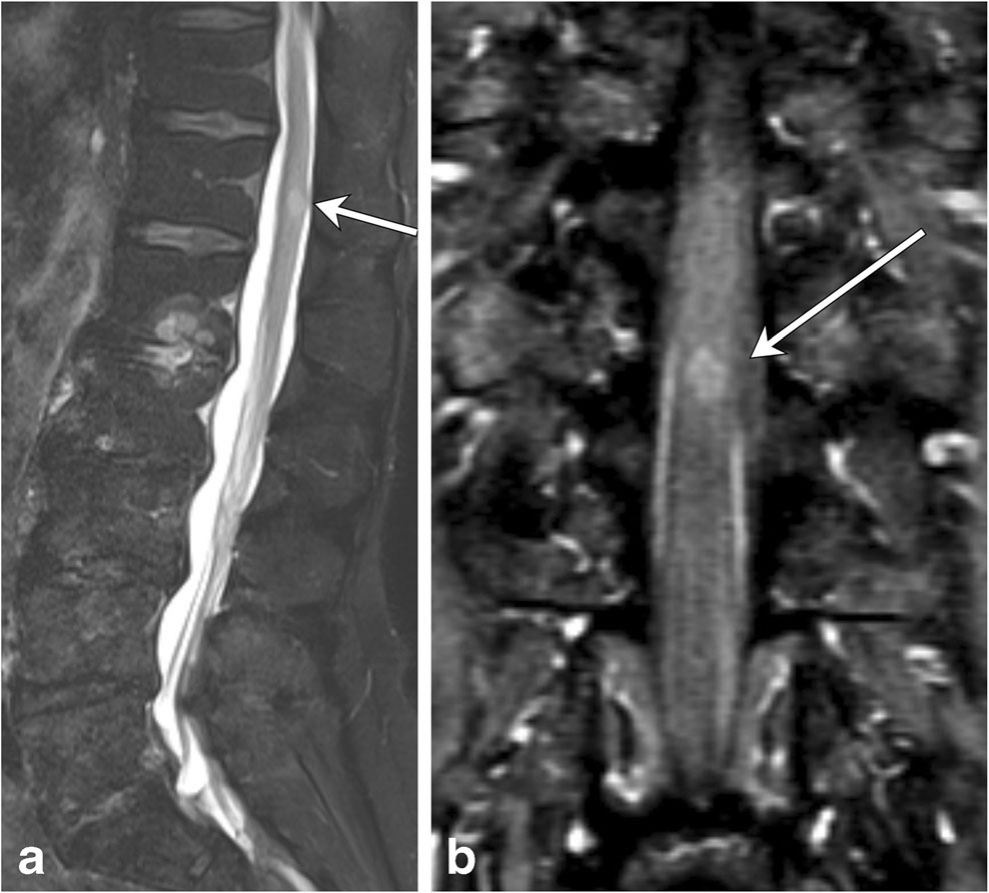
Spinal Cord Metastasis. A 69-year-old male with a history of small cell lung cancer and new back pain. **a** There is a focus of intramedullary T2 hyperintensity (arrow), which enhances (**b**, arrow) on the T1 post-contrast coronal image

**Fig. 17:**
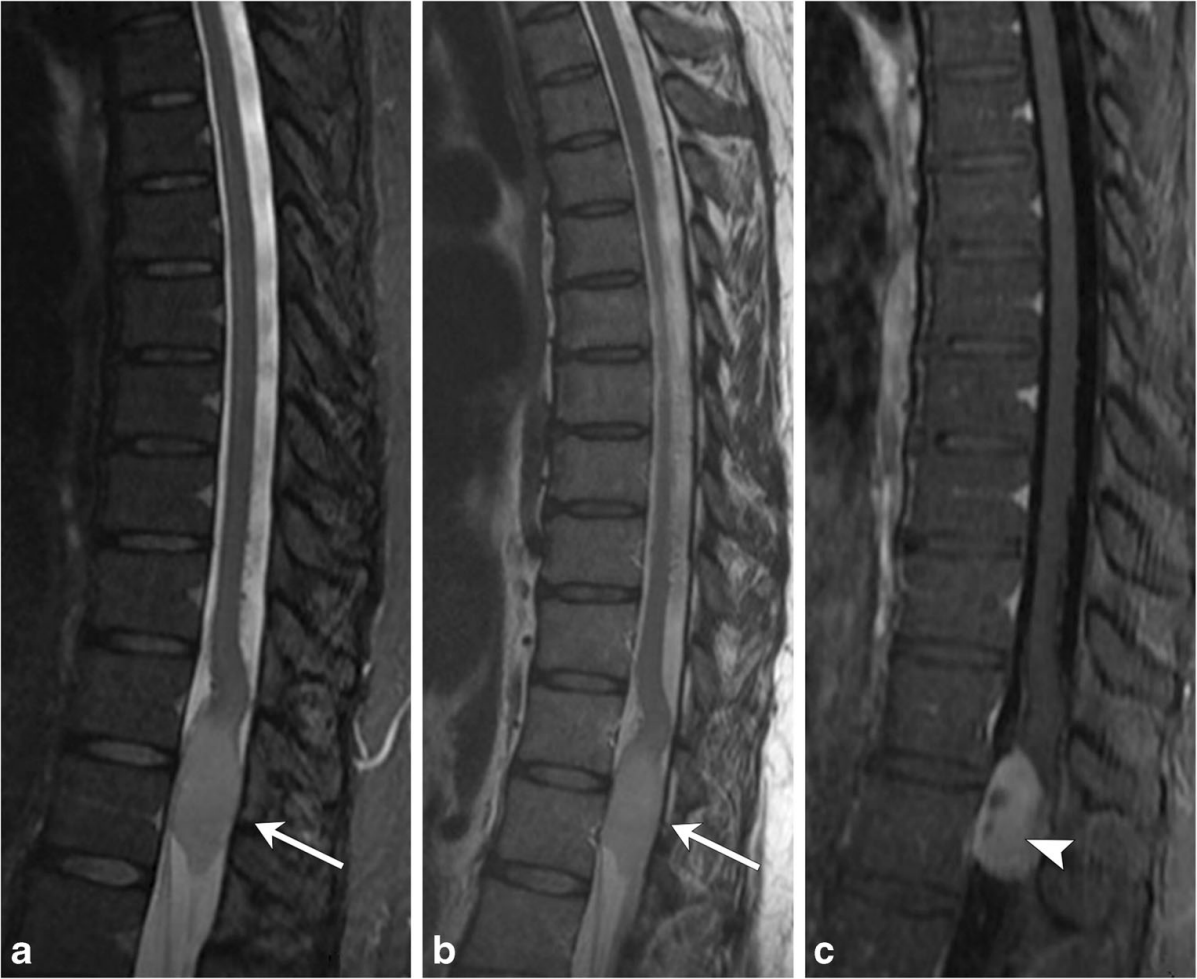
Glioblastoma multiforme. A 46-year-old female with lumbar back pain and recent onset of urinary retention. There is an expansile lesion within the distal spinal cord with intermediate T2 hyperintensity (**a**, arrow) and non-uniform enhancement (**c**, arrowhead)

In intramedullary neoplasms, the length of involvement, location along the cord, and enhancement pattern are the most helpful in narrowing the differential diagnosis (Table 3). Spinal cord astrocytomas will involve a long segment, as will (but to a lesser extent) ependymomas, while hemangioblastomas and metastases typically involve short segments. The enhancement pattern is essential in differentiating these lesions. In ependymoma, a solid, mostly homogeneous pattern of enhancement is typical, while astrocytomas will exhibit more heterogeneous enhancement. Other distinguishing features of ependymoma include presence of tumoral and nontumoral cysts and identification of a “cap sign”, which is a rim of T2 hypointense hemosiderin secondary to associated hemorrhage that can occur in about one-third of cases [24]. More aggressive subtypes, such as the rare spinal cord glioblastoma multiforme, tend to exhibit areas of hemorrhage and non-uniform enhancement (Fig. 16). In hemangioblastomas, presence of a feeding vessel, detected as a flow void on T2-weighted images, and a focus of intense nodular enhancement are characteristic findings. When multiple and short segments of enhancement are involved, metastases should be considered (Fig. 17), and search for a primary malignancy should ensue if not already known.

**Table 3:** Neoplastic lesion summary

Neoplastic lesions	Location	Length and segment distribution	Enhancement
Astrocytoma	Thoracic > cervical	4–7 Vertebral bodies	Heterogeneous
Ependymoma	Cervical > thoracic	4 Vertebral bodies	Solid Heterogeneous
Hemangioblastomas	Thoracic > cervical Eccentric, may be exophytic	Short segment	Nodular Feeding vessel
GBM	Nonspecific	Nonspecific	Irregular Heterogeneous
Metastasis	Cervical > thoracic > lumbar	Multiple Several segments	Small compared to extent of edema

## Vascular lesions

### Dural arteriovenous fistulas

**Fig. 18:**
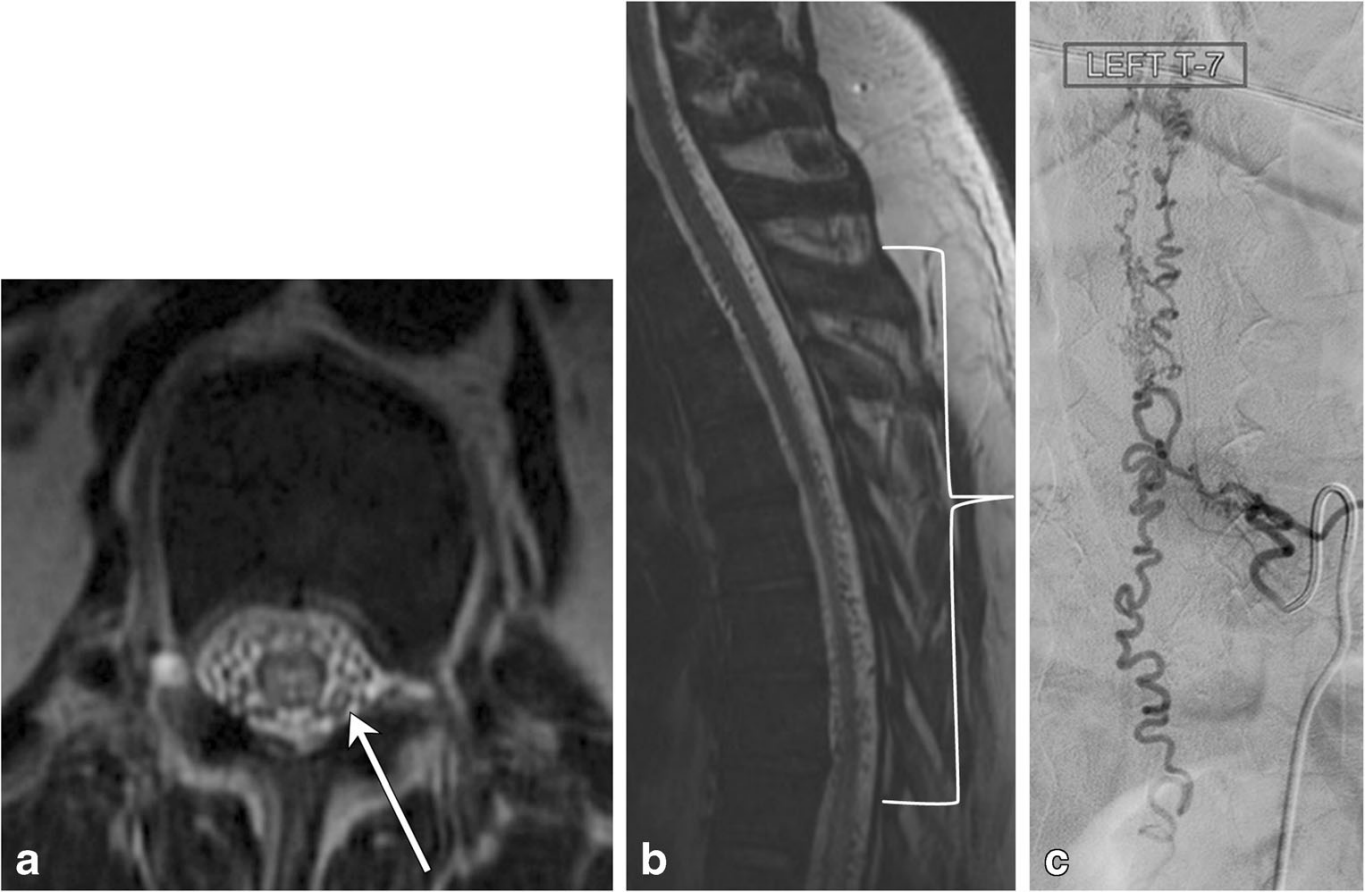
Dural arteriovenous fistula. A 50-year-old male with gradual onset of inability to walk, off and on constipation, and urinary incontinence. **a** Axial T2 image demonstrates hyperintensity in the cord and multiple serpiginous flow voids (arrow) within the thecal sac. **b** Sagittal T2 image reveals long segment involvement (bracket). **c** Conventional spinal angiogram shows injection of a paraspinal artery with simultaneous robust filling of a corkscrew venous vessel

**Fig. 19:**
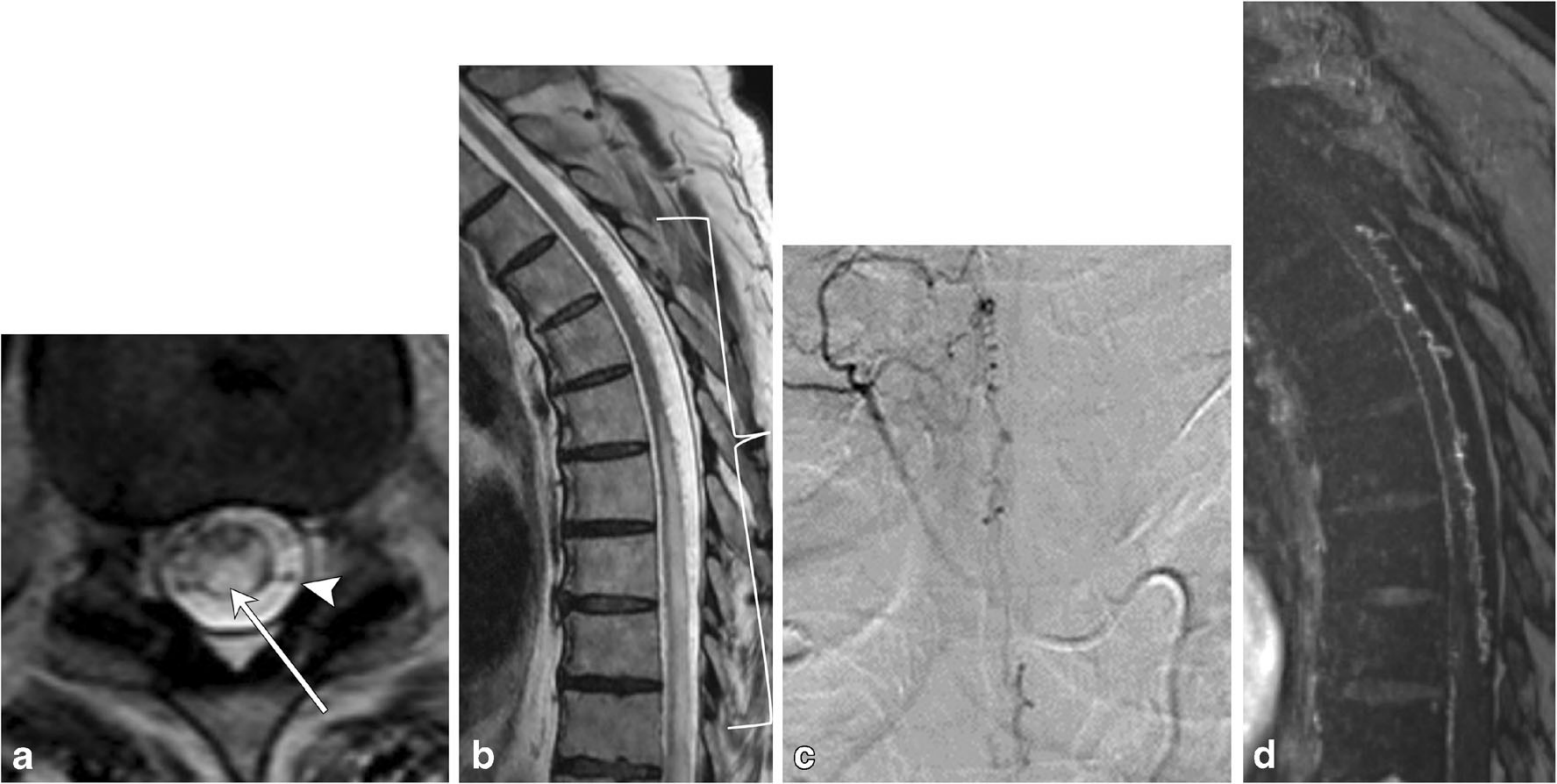
Dural arteriovenous fistula. A 72-year-old male with progressive lower extremity weakness, without improvement in symptoms after lumbar laminectomy. **a** Axial T2 image demonstrates numerous flow voids in the thecal sac (arrowhead). There is high signal in the cord due to congestion (arrow). **b** There is long-segment high T2 signal in the cord, with serpiginous vessels in the posterior thecal sac (bracket). **c** Spinal angiogram shows the right T4–T5 supreme intercostal artery as a feeding vessel. **d** MRA confirms the arteriovenous connection

Dural arteriovenous fistulas (dAVF) are the most common spinal cord vascular malformation (Figs. 18 and 19). These lesions are composed of a direct communication between a nerve root sleeve dural artery and vein and are categorised as a type I spinal vascular malformation. They are usually diagnosed late because of their prevalence in the elderly male population and nonspecific symptomatology, which often overlap with those of spondylosis or polyneuropathy [25]. A common pattern of diagnosis is in those with post-laminectomy syndrome, who exhibit persistent symptoms after spinal surgery for spondylosis. Although digital subtraction angiography is the gold standard study for definitive diagnosis, MRI and magnetic resonance angiography (MRA) techniques have become increasingly more sensitive for detection of this rare, but important spinal pathology. On MRI, serpentine flow voids on the surface of the cord with engorged venous structures and intramedullary hyperintensity on T2-weighted sequences are reliable clues to the diagnosis [26, 27]. The condition should be considered in patients with insidious myelopathy, as it can be reversible if diagnosed and treated early in the course of the disease.

### Arteriovenous malformations

**Fig. 20:**
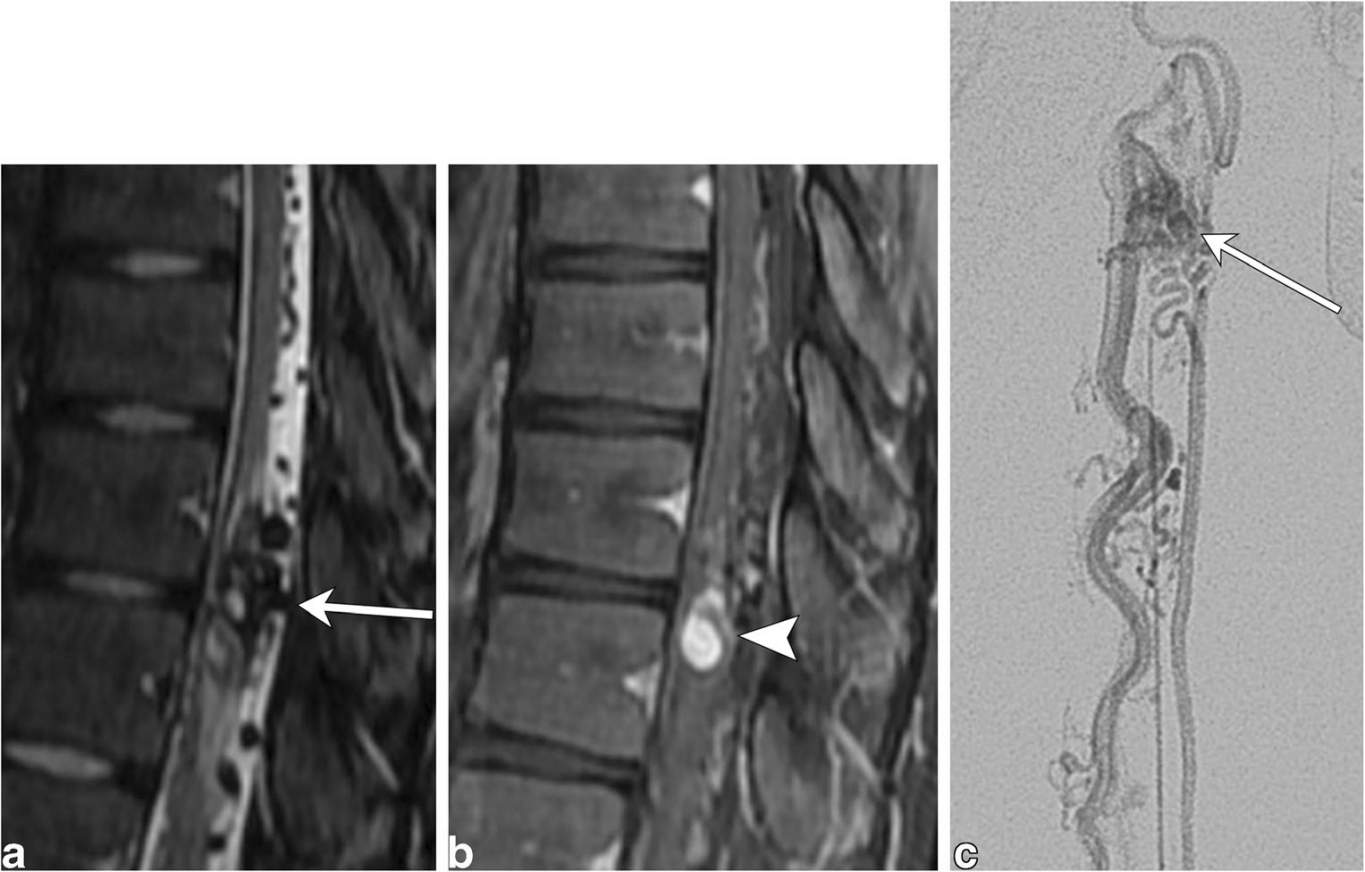
Arteriovenous malformation. A 16-year-old male with left leg weakness and progressive areflexia. **a** Sagittal T2 fat-saturated image demonstrates a conglomeration of flow voids within the cord parenchyma with surrounding hyperintensity. Prominent extramedullary flow voids lead to this conglomeration of vessels (arrow). **b** The sagittal T1 post-contrast image shows a nodule of enhancement (arrowhead). **c** Conventional angiogram shows an artery feeding a nidus (arrow), which is drained by a prominent vein

Intramedullary spinal arteriovenous malformations (AVM) are significantly rarer than dAVFs, representing about 25% of vascular lesions within the spine. They can present as a spinal subdural hemorrhage or progressive myelopathy in which patients initially experience spastic paralysis followed by flaccid paralysis of the extremities, accompanied by sensory deficits and sphincter dysfunction. This clinical presentation has been termed the Foix-Alanjouanine syndrome [28]. Spinal arteriovenous malformations contain a true arteriovenous capillary nidus fed by an enlarged feeding artery and drained via an enlarged venous plexus on the cord surface (Fig. 20). They are categorised as type II spinal vascular malformations and most commonly occur within the cervical or thoracic cord [28–31].

### Cavernoma

**Fig. 21:**
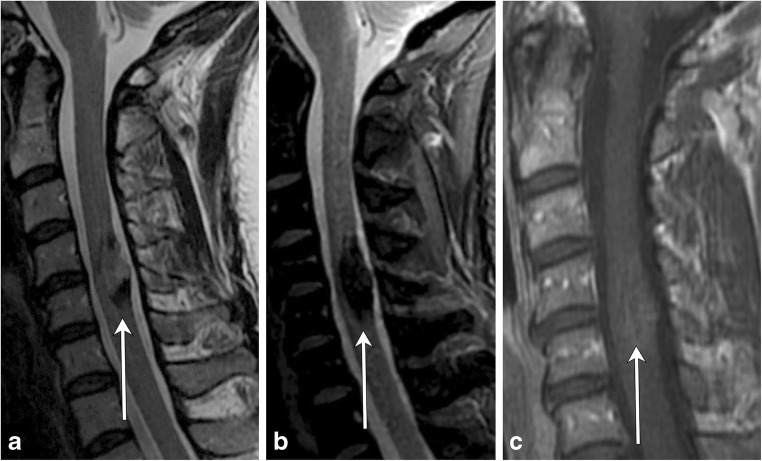
Cavernoma. A 40-year-old female with left upper extremity numbness. **a** Sagittal T2 image shows a short segment mixed signal lesion and layering of low signal at the inferior aspect. **b** The lesion is hypointense on the sagittal GRE image. **c** Sagittal T1 post-contrast image shows minimal strands of enhancement

Most commonly found in the thoracic cord, spinal cavernomas (also known as cavernous malformations) are rare lesions that commonly present intracranially. They consist of endothelial-lined lacunae filled with blood and surrounded by thick walls. They are occult on angiography as they do not communicate with the cord vasculature and have similar imaging characteristics as their intracranial counterparts [32, 33] (Fig. 21).

### Infarct

**Fig. 22:**
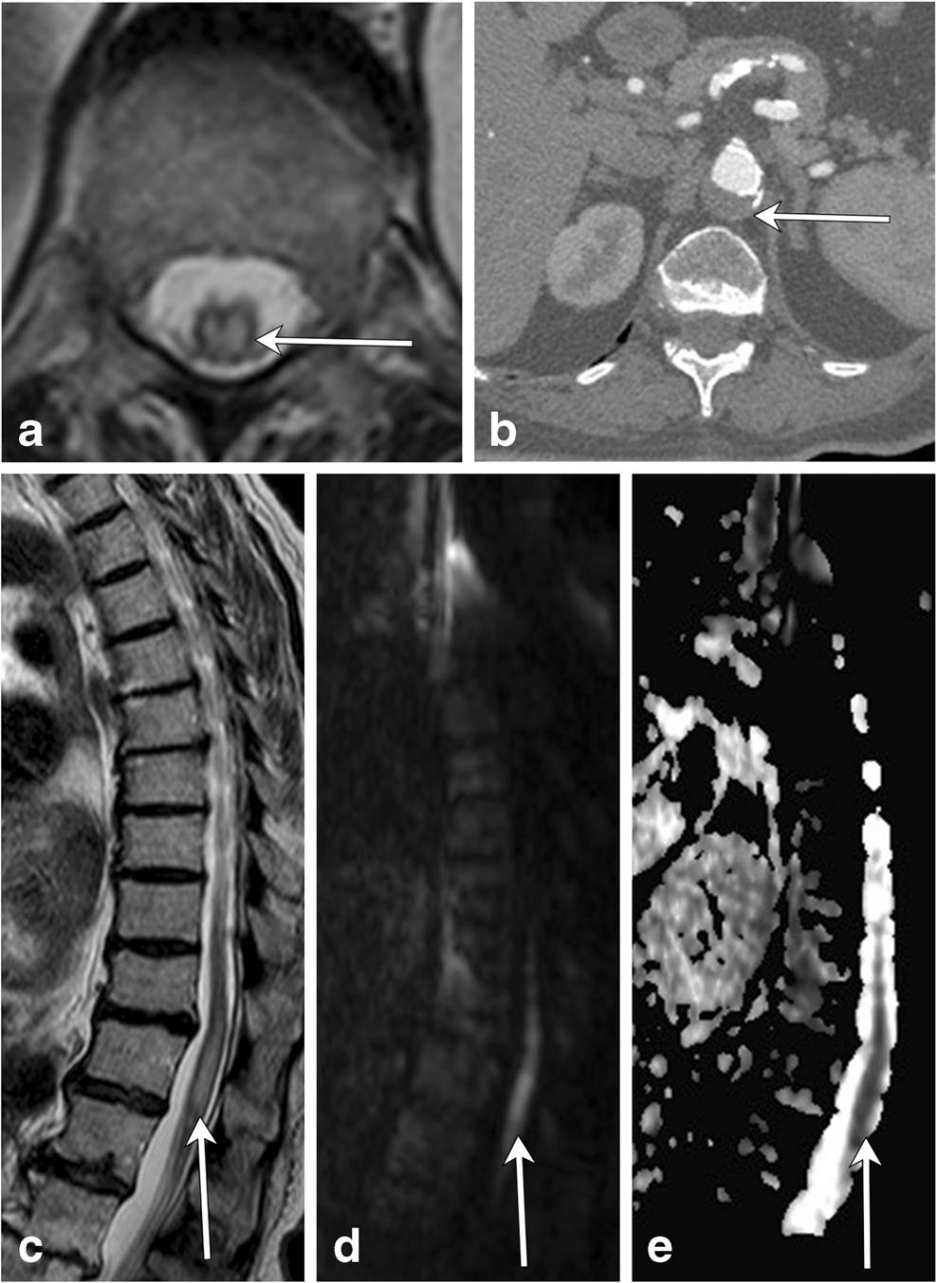
Spinal cord infarction. An 82-year-old female with hypertension and hyperlipidaemia presents with acute bilateral lower extremity weakness. **a** Axial T2 image demonstrates a large cross-sectional area of hyperintensity (arrow) within the thoracic cord. **b** Axial CT angiogram image shows mural thrombus within the aorta (arrow) at the same level of the spinal cord findings with no appreciable filling of the lumbar arteries. Sagittal T2 (**c**), DWI (**d**) and ADC (**e**) images reveal a long-segment intramedullary lesion in the lower cord (arrows) that is T2 hyperintense and has restricted diffusion

Spinal cord infarctions occur very rarely, accounting for less than 1% of strokes. However, appropriate diagnosis of this entity is critical, as its symptoms overlap with other causes of myelopathy, such as demyelinating processes. There are many underlying aetiologies of spinal cord infarction, and they are classified according to the transverse involvement of the cord, which corresponds to the insulted vascular territory. MRI is the gold standard for diagnosis. All cases involve a long segment of the cord, and the majority of infarcts will involve the anterior spinal artery, resulting in anterior or central cord involvement [34] (Fig. 22).

### Distinguishing vascular lesions

Segment length involvement is a very important distinguishing factor in spinal vascular lesions. Long-segment cord T2 hyperintensity should raise the suspicion of a dAVF or spinal cord infarct, while short segment involvement raises the possibility of a cavernoma or arteriovenous malformation. Cavernous malformations usually do not cause cord edema or expansion. “Popcorn-like” heterogeneity from blood products of differing ages, a hemosiderin perimeter (low T2 signal rim), and susceptibility or blooming on gradient echo sequences are classic findings. DAVFs almost always demonstrate T2 hyperintensity at the conus medullaris because of dependent cord edema, and identification of serpiginous flow voids on the surface of the cord is immensely helpful in making the diagnosis. This is in contrast to finding intramedullary serpentine flow voids and a short segment of intramedullary signal change, which should point to the presence of a nidus in an AVM. A long segment “owl-eye” appearance of bilateral ventral horn hyperintensity on T2-weighted images is concerning for the presence of anterior spinal artery infarction and should prompt a search for a possible underlying aetiology, such as severe atherosclerotic aortic disease, an aortic dissection or aneurysm (Table 4). Although rarely present, signal changes in the adjacent vertebral body manifesting as marrow edema or osseous infarction can be helpful in confirming the diagnosis of a spinal cord infarct [35].

**Table 4:** Vascular lesions summary

Vascular lesions	Location	Length and segment distribution	Enhancement
Arteriovenous malformation	Dorsal Lower thoracic cord and conus medullaris	Short segment	Heterogeneous, nodule of enhancement
Dural arteriovenous fistula	Variable Edema of lower thoracic cord and conus	Long segment	+/− Enhancement
Cavernoma	Thoracic > cervical	Short segment	Minimal, heterogeneous
Infarction	Depends on territory, anterior cord most common	Long segment	Depends on infarct age

## Miscellaneous

### Compressive myelopathy

**Fig. 23:**
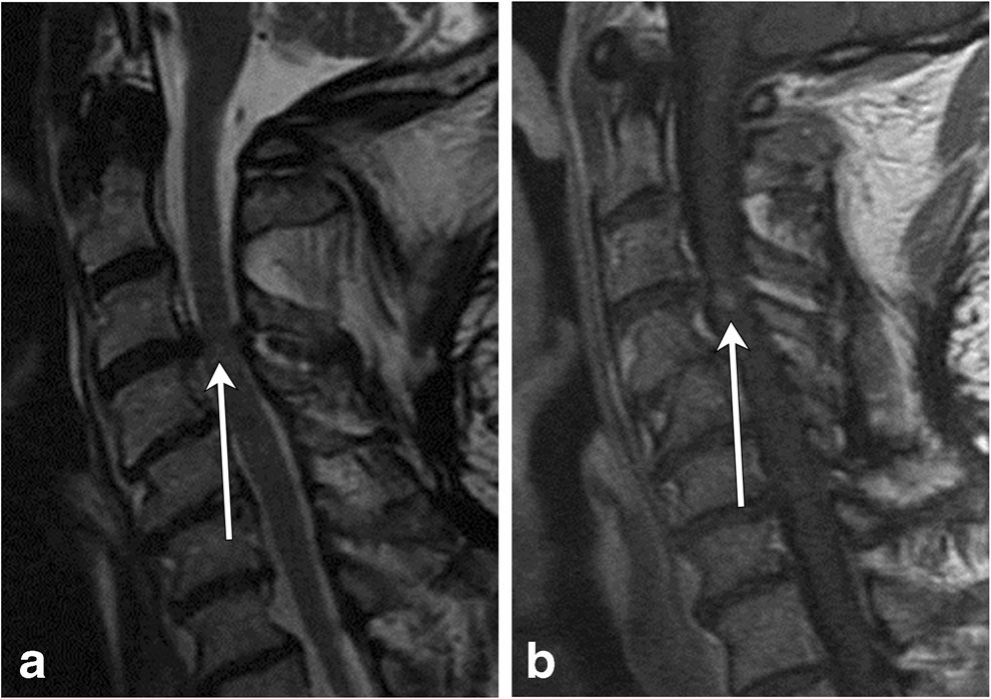
Compressive myelopathy. A 50-year-old patient with bilateral hand tingling. **a** Sagittal T2 image shows severe spinal canal narrowing at C3–C4 due to degenerative changes, with focal faint signal hyperintensity (arrow). **b** Post-contrast image shows focal enhancement (arrow)

A common cause of weakness and sensory deficits in older patients is cervical compressive myelopathy or myelopathic spondylosis, which can occur secondary to spondylotic changes in the cervical spine and less commonly because of congenital abnormalities. The mechanism of injury involves decreased canal diameter and spinal stenosis, with resultant focal signal abnormality within the affected cord (Fig. 23). Ossification of the posterior longitudinal ligament (OPLL) is a common contributor. Compressive myelopathy typically demonstrates focal T2 hyperintensity in the cord at a level of spinal stenosis (at the level of a disc) with or without associated enhancement [36, 37] (Table 5).

**Table 5:** Miscellaneous lesion summary

Miscellaneous	Location	Length and segment distribution	Enhancement
Compressive myelopathy	At the disc level Cervical	Focal, short segment	May see focal enhancement
Hirayama disease	Anterior horns Cervical	Long segment	May see enhancement of enlarged epidural space

### Hirayama disease

**Fig. 24:**
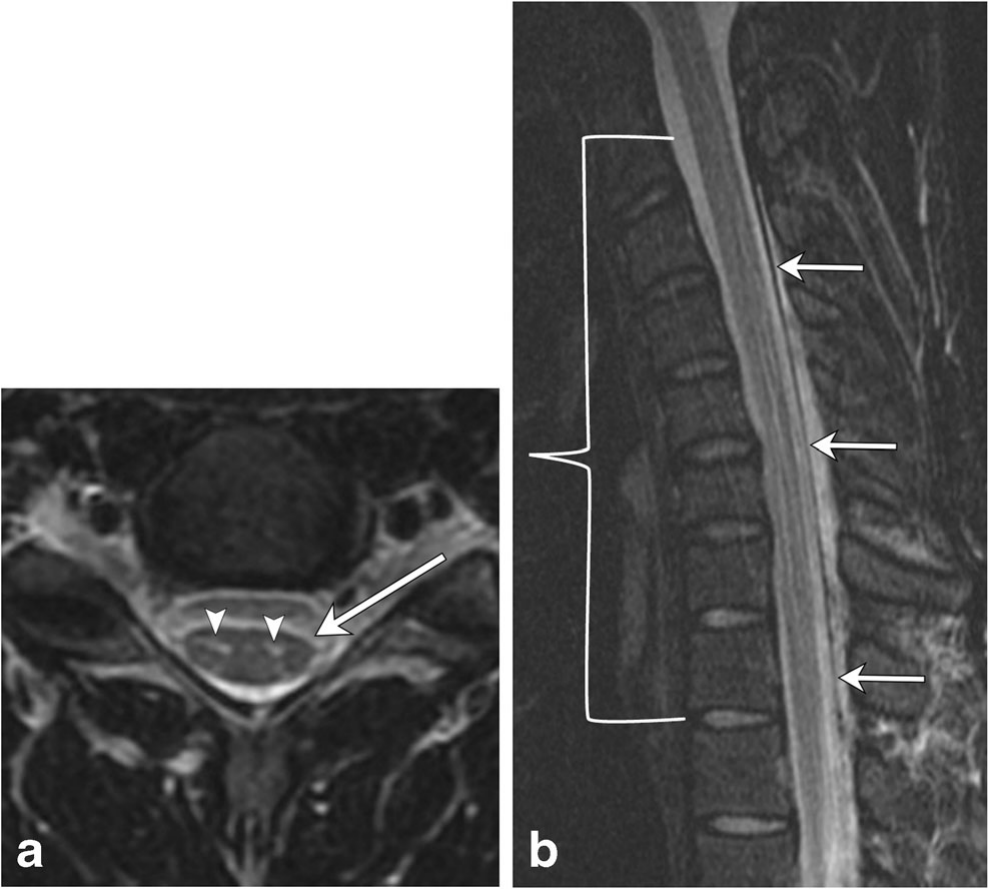
Hirayama disease. A 22-year-old male with upper extremity weakness and muscle atrophy. **a** There is focal T2 hyperintensity in the anterior horns (arrowheads). Subtle asymmetric focal cord atrophy at this level (C5–C6), slightly more conspicuous on the left (arrow). **b** Flexion view shows anterior displacement of the dura (arrows) with narrowing of the thecal sac (bracket), extending from C3 to T2, most pronounced at C5–C6. Note the long segment T2 hyperintensity within the anterior horns

Hirayama disease is a disease of young males, mostly recognised in Japan and India. It presents with unilateral upper extremity weakness. The disease is related to a lax dura mater, which changes position with flexion and extension. Resulting repetitive circulatory changes in the anterior spinal artery due to cord compression cause atrophy and eventual necrosis in the anterior horns. MRI plays an essential role in the diagnosis of this rare disease (Fig. 24). Neutral cervical spine images are not very sensitive in showing the pertinent changes, and if there is clinical concern for this condition, flexion images should be obtained. Forward displacement of the dura mater is seen, with anterior cord compression and enlargement of the posterior epidural space, which may enhance post contrast. On transverse sections, there is atrophy of the anterior horns, which is typically asymmetric [38, 39] (Table 5).

## Conclusion

As seen, there are myriad causes of abnormal signal within the spinal cord; demyelinating, metabolic, neoplastic, and vascular aetiologies must be considered when evaluating an abnormal cord. Although intramedullary spinal cord pathology can be a challenge, a systematic approach to imaging interpretation of these conditions focused on the length, location, and pattern of enhancement can greatly narrow the differential diagnosis, if not arrive at the correct diagnosis. This will not only add value to the care of the patient, but has the potential to prevent the use of more invasive methods of diagnosis.
